# Katatones Wandern mit Emil Kraepelin, Aloys Alzheimer, Henry Aloysius Cotton und anderen 

**DOI:** 10.1007/s00115-020-01039-z

**Published:** 2020-12-16

**Authors:** Hans Förstl

**Affiliations:** Klinik und Poliklinik für Psychiatrie und Psychotherapie, TU München, Ismaningerstr. 22, 81675 München, Deutschland

**Keywords:** Evidenz-basierte Medizin, Nationalismus, Noxentheorie, Rassenhygiene, T4, Evidence-based medicine, Nationalism, Theory of noxious agents, Racial hygiene, T4

## Abstract

Die Betriebsausflüge von Emil Kraepelins Königlich Psychiatrischer Klinik wurden unter den Mitarbeitern als „katatone Wanderungen“ bezeichnet. Im Jahr 1906 nahm eine erstaunliche Zahl deutscher und internationaler Gäste daran teil, Nicolas Achucarro, Henry Cotton, Eduard Flatau, Smith Ely Jelliffe, Gaetano Perusini, Edward Scripture, Maurycy Urstein und andere. Viele von Kraepelins Kollegen waren inspiriert von seinen Ideen und getrieben von wissenschaftlichem Enthusiasmus, der neben fachlichen Fortschritten in der Nervenheilkunde zu ganz unterschiedlichen Ergebnissen führte, von der Zahn- und Bauchchirurgie zu Psychoanalyse und Evidenz-basierter Medizin; von Rassenhygiene und Nationalismus zur Präsidentschaft des kommunistischen Rumänien.

## Abstract (en)

Staff outings of Emil Kraepelin’s Royal Department of Psychiatry were known as “catatonic walks”. A remarkable number of important German and international visitors participated in 1906, Nicolas Achucarro, Henry Cotton, Eduard Flatau, Smith Ely Jelliffe, Gaetano Perusini, Edward Scripture, Maurycy Urstein and others. Many of Kraepelin’s collaborators were inspired by his ideas and driven by scientific enthusiasm which contributed to significant scientific advances, but also took them to very different ends from dental and bowel surgery to psychoanalysis and eventually evidence-based medicine; from racial hygiene and nationalism to the presidency of communist Romania.

Nach Eröffnung der Königlich Psychiatrischen Klinik München im Jahr 1904 war die Personalausstattung zunächst übersichtlich. So werden im Amtlichen Verzeichnis des Personals im Sommersemester 1906 Emil Kraepelin als Direktor und Robert Gaupp als Oberarzt aufgeführt, Hans Gudden als Leiter der Poliklinik, Aloys Alzheimer als wissenschaftlicher Assistent sowie vier klinische Assistenzärzte Paul Nitsche (1. Assistent), Karl Weiler (2. Assistent), Otto Rehm (3. Assistent) und Alfred Busch (4. Assistent). Daneben erwähnt werden drei interne und zwei externe, namentlich nicht bezeichnete Volontärärzte [14]. Dabei handelte es sich um Otto Gross, Werner Lüttge und Felix Plaut sowie die Studenten Carl Bastin, Eduard Reiß, Paul Weber und den Offizier Palmberger [6, 8, 17]. Umso überraschender ist die internationale Beteiligung am Betriebsausflug im Sommer 1906, bei dem Smith Ely Jelliffe aus den USA dieses Gruppenbild machen ließ (Abb. 1). Laut Jelliffe wurden diese einmal jährlich stattfindenden Ereignisse von Mitarbeitern als „katatone Wanderungen“ bezeichnet [13].

**Figure 1:**
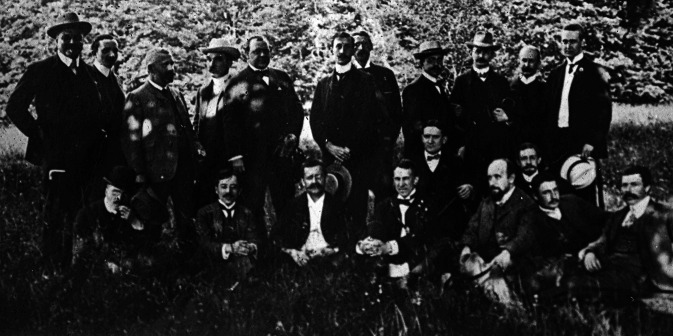

Jelliffe reiste gemeinsam mit Pearce Bailey und die beiden arbeiteten im Sommersemester 1906 sechs Monate an der Klinik mit Kraepelin und im Labor mit Alzheimer [4, 13]. Er betonte in seinem Beitrag den Enthusiasmus der Assistenten und Forscher einschließlich der internationalen Gäste, von denen er Scripture und Cotton aus den USA, Parhon aus Rumänien, Sibelius aus Finnland und Lundborg aus Schweden namentlich erwähnt [4, 13]. Die drei letztgenannten finden sich nicht auf dem Ausflugsfoto und ebenso wenig Weiler und Nitsche, der unter einer länger dauernden Erkrankung litt [19].

Diese Konzentration vielversprechender und einflussreicher Nervenärzte auf einer sommerlichen Wanderung ist bemerkenswert. Die Teilnehmer haben die Nervenheilkunde im Positiven und Negativen stark geprägt. Einige waren gerade berufen worden (Flatau in Warschau, Gaupp nach Tübingen) oder auf dem Weg zu einer neuen und einflussreichen Position. Tab. 1 verrät womit sich Kraepelin und seine Kollegen in dieser Zeit wissenschaftlich beschäftigten.

**Table 1:** 

Name, Vorname		H	Veröffentlichungen aus dem Jahr 1906 ff. z. B.
Achucarro, Nicolas	1880–1918	Sp	(Diss.) Contribucion al estudio de la anatomia patologica de la rabia
Allers (Abeles), Rudolf	1883–1963	A	(BB) Aufbau des Chemischen Laboratoriums; in [18]
Alzheimer, Aloys	1864–1915	D	(Z) Über einen eigenartigen schweren Erkrankungsprozess der Hirnrinde [1]
Bailey, Pearce	1865–1922	US	(B) Diseases of the Nervous System Resulting from Accident and Injury
Busch, Alfred	1876–	D	(Z, 1910, mit F. Plaut) Über die Einwirkung verlängerter warmer Bäder auf einige körperliche und geistige Funktionen
Cotton, Henry A	1876–1933	US	(Z, 1915^b^) Fatty degeneration of the cerebral cortex in the psychoses with special reference to dementia praecox [7]
Flatau, Eduard	1868–1932	P	(B, 1912) Die Migräne
Gaupp, Robert	1870–1953	D	(Z) Die klinischen Besonderheiten der Seelenstörungen unserer Grossstadtbevölkerung
Gross, Otto^a^	1877–1920	A	(B) Das Freudsche Ideogenitätsmoment und seine Bedeutung im manisch-depressiven Irresein Kraepelins
Gudden, Hans	1866–1940	D	(Z) Die Zurechnungsfähigkeit bei Warenhausdiebstählen
Jelliffe, Smith Ely	1866–1945	US	Unermüdlicher Autor und Hrsg z. B. von J. Nervous & Mental Disease; Psychoanalytic Review, u. v. a.
Kirby, George Hughes^a^	1875–1935	US	(Z) The psychiatric clinic at Munich, with notes on some clinical psychological methods
Kraepelin, Emil	1856–1926	D	(B) Über Sprachstörungen im Traume; (Z) das Verbrechen als soziale Krankheit; der Alkoholismus in München; über hysterische Schwindler
Lundborg, Hermann^a^	1868–1943	Sch	(Z, 1912) Der Erbgang der progressiven Myoklonusepilepsie
Nitsche, Hermann Paul^a^	1876–1948	D	(Z, 1907) Über chronische Manie
Parhon, Constantin I.^a^	1874–1969	R	(B, 1909, mit M. Goldstein) Les Secretions Internes: Pathologie et Physiologie
Perusini, Gaetano	1879–1915	I	(Z) Tabes dorsalis, Tabesparalyse oder Myelitis
Plaut, Felix^a^	1877–1940	D	(BB) Progressive Paralyse; Serodiagnostik; in [17]
Probst, Ferdinand	1867–1923	A	(B, 1904!) Der Fall Otto Weininger
Rehm, Otto	1876–1941	D	(B, mit F. Plaut 1913^b^) Leitfaden zur Untersuchung der Zerebrospinalflüssigkeit
Rohde, Erwin	1881–1915	D	(Diss, 1908) Stoffwechselversuche bei Epileptikern
Scripture, Edward W	1864–1945	US	(B) Researches in Experimental Phonetics: the Study of Speech Curves
Sibelius, Christian^a^	1869–1922	F	(Z) Zur Kenntnis der Zweiteilung des Rückenmarks
Urstein, Maurycy	1872–1940	P	(Z) Ein Beitrag zur vergleichenden Psychiatrie
Weiler, Karl^a^	1878–1973	D	(BB) Warmwasserbehandlung; Dementia praecox; Unfallfolgen; in [17]

*A* Österreich; *F* Finnland; *H* Herkunftsland; *I* Italien; *R* Rumänien; *P* Polen; *Sch* Schweden; *Sp* Spanien; *US* Vereinigte Staaten

*B* Lehrbuch; Monographie; *BB* Buchbeitrag; *Diss* Dissertation; *Z* Zeitschriftenbeitrag

^a^Nicht auf Abb. 1

^b^Diese später publizierten Arbeiten wurden nach Angabe der Autoren in München begonnen

Achucarro verteidigte 1906 seine Dissertation über die Pathologie der Tollwut erfolgreich in Madrid. Allers betätigte sich pflichtbewusst im Chemielabor, wandte sich aber Jahre später in den USA der Psychotherapie und Philosophie zu. Alzheimer hatte im April 1906 das Gehirn von Auguste D. untersucht und hielt am 3. November des Jahres seinen gleichermaßen bescheidenen wie bahnbrechenden Vortrag über die Grundlagen der präsenilen Demenz [1, 11]. Gaupp, Gudden und Kraepelin äußerten sich zu den psychischen Problemen im modernen München und vielen anderen Themen. Flatau, Urstein und Sibelius (der jüngere Bruder des Komponisten) gründeten Fachgesellschaften in ihren Heimatländern. Alle in Tab. 1 aufgeführten Forscher waren nach 1906 wissenschaftlich produktiv geblieben und die meisten haben sich in der wissenschaftlichen Literatur nachhaltig verewigt. Einige können sogar Eponyme für sich reklamieren, z. B. die Alzheimer-Erkrankung (in Italien Perusini-Alzheimer-Erkrankung; [5]), von Economo-Flatau-Erkrankung (in Polen für Encephalitis lethargica), Flatau-Redlich-Krankheit (Encephalomyelitis epidemica disseminata), Flatau-Sterling-Dystonie (Torsionsspasmus bei Kindern), Flatau-Gesetz (die exzentrische Lagerung der langen Bahnen im Rückenmark), Kraepelin-Bleuler-Erkrankung (Schizophrenie), progressive Myoklonusepilepsie Unverricht-Lundborg, Parhon-Syndrom der inadäquaten Sekretion von antidiuretischem Hormon (SIADH) oder das Urstein-Stransky-Syndrom der „intrapsychischen Ataxie“ bei Schizophrenie.

Das Netzwerk war international, fachlich vielfältig und wurde ständig erweitert [10]: Bailey, Cotton, Jelliffe, Scripture kehrten in die USA zurück, Achucarro und Allers folgten, auch Kraepelin besuchte später die Neue Welt. Jelliffe kam zu weiteren Besuchen nach Europa, Scripture und Achucarro kamen zurück, um zu bleiben und ihre Forschung auszubauen. Alzheimer, Achucarro und Perusini verstarben früh. Rohde verlor im 1. Weltkrieg das Leben. Felix Plaut emigrierte 1935 nach London.

Mehr als 3000 Hochschullehrer des Deutschen Reiches hatten am 23.10.1914 die berüchtigte Erklärung unterschrieben: „… der Dienst im Heere macht unsere Jugend tüchtig auch für alle Werke des Friedens, auch für die Wissenschaft …“ [9]. Dazu gehörten auch Kraepelin, Gudden, Busch und der später in die Klinik eingetretene Walter Spielmeyer. Völkische Gesinnung war bei einigen mit rassenbiologischen Überzeugungen gepaart. Gaupp, aber auch Bailey und vor allem Lundborg profilierten sich als Rassenhygieniker. Nitsche wurde eine treibende Kraft der Aktion T4 [3]. Ungewöhnlich war die Entwicklung des radikalen Sozialisten Parhon, der nicht nur Psychiatrie und Endokrinologie, sondern die gesamte Medizin und Politik des kommunistischen Rumänien wesentlich beeinflusste; er war von 1947 bis 1948 Präsident des Provisorischen Präsidiums und von 1948 bis 1952 der erste „Präsident des Präsidiums der Nationalversammlung“ (Staatspräsident). Karl Weiler wurde nach dem Krieg immerhin Präsident der Bayerischen Landesärztekammer und überlebte alle anderen (Tab. 1; [2]).

Eine ganz besondere Bedeutung erlangte Henry Aloysius Cotton (Abb. 1, hinter der ersten Reihe kniend; [7]). Wenige Woche nach der Wanderung in Oberbayern übernahm er mit 31 Jahren die Position als Superintendent des heruntergekommenen Trenton State Hospital in New Jersey. Er war ein energischer Reformer, führte Nonrestraint und moderne medizinische Behandlungsmethoden ein (z. B. Salvarsan und Hydrotherapie), war aber nach zehn Jahren enttäuscht von den vergleichsweise bescheidenen Erfolgen in der Psychiatrie. Unter dem Eindruck der bakteriologischen Revolution und weiterhin inspiriert von Kraepelins Noxentheorie begann er die vermeintlichen Giftherde aus dem Körper seiner Patienten zu entfernen, zog in den Jahren 1919 bis 1920 mehr als 4000 und im Jahr 1921 bereits 6000 Zähne [12, 20]. Die meisten seiner Patienten wurden auch tonsillektomiert. Da dies meist nicht genügte, um eine befriedigende Heilung herbei zu führen, nahm er größere Eingriffe in der Bauchhöhle vor und entnahm Teile des Kolons („Weisheitszahn der Gedärme“) aus dem linken und rechten unteren Quadranten [12, 20]. Die Heilungsraten waren angeblich enorm, führten zu sensationellem Ruf und Zulauf. Bereits die erste oberflächliche Ordnung der Patientenakten durch Phyllis Greenacre bewies jedoch, dass die Sterblichkeit bei den großen Eingriffen größer war als die reklamierten Erfolge [12]. Die Veröffentlichung dieser kritischen Untersuchung wurde von Adolf Meyer, dem früheren Mentor Cottons, lange Zeit verhindert [21]. Die Affäre führte aber zu einer der ersten interdisziplinären, systematischen, kontrollierten Studien [15, 16, 22], die von George Kirby initiiert wurde, der 1906 ebenfalls einige Monate bei Kraepelin in München verbracht hatte [8].

Die Stimmung wissenschaftlichen Aufbruchs und der Wille zum Durchbruch von der Theorie in die medizinische Praxis und bis in die Gesellschaft hinein trugen zu katastrophalen Konsequenzen bei, die sich nicht auf Deutschland beschränkten. Einige der in Abb. 1 abgebildeten Männer tragen damit eine historische Verantwortung, die sie damals nicht ahnen konnten. Ethik und evidenzbasierte Medizin bilden heute gerade aufgrund dieser Entwicklungen ein Gegengewicht zu Ideen und Projekten deren potenzielle Folgen begeisterte Forscher nicht immer abschätzen können.
